# T174M-M235T *AGT* Gene Haplotypes in Women with Pre-Eclampsia from Northwest Mexico: A Pilot Case-Control Study

**DOI:** 10.3390/cimb48020168

**Published:** 2026-02-02

**Authors:** Jorge H. Portillo-Gallo, Jorge Manuel Sánchez-González, Ana Miriam Saldaña-Cruz, Martha Rocío Hernández-Preciado, Luis Arturo Camacho-Silvas, Verónica Michelle Ledesma-Martínez, Héctor Alfonso Gómez-Rodríguez, Jhonathan Cárdenas-Bedoya, Ingrid Patricia Dávalos-Rodríguez, Rafael Franco-Santillán, María Cristina Morán-Moguel

**Affiliations:** 1 Instituto Nacional de Aprendizaje, Habilidades e Investigación de las Ciencias (INAHIC), Diana Natura H1, Zapopan ZC 45221, JA, Mexico; galeno_100@hotmail.com (J.H.P.-G.); juevesm@gmail.com (J.M.S.-G.); 2 Federación Mexicana de Patología Clínica y Medicina de Laboratorio, Av. Cuauhtémoc 330, Colonia Doctores ZC 06720, CDMX, Mexico; 3 Instituto de Terapia Experimental y Clínica (INTEC), Departamento de Farmacología, Centro Universitario de Ciencias de la Salud, Universidad de Guadalajara, Sierra Mojada 950, Colonia Independencia, Guadalajara ZC 44340, JA, Mexico; ana.saldanac@academicos.udg.mx; 4 Departamento de Disciplinas Filosófico, Metodológicas e Instrumentales, Centro Universitario de Ciencias de la Salud, Universidad de Guadalajara, Sierra Mojada 750, Puerta 1B Edificio N, Colonia Independencia, Guadalajara ZC 44340, JA, Mexico; martha.hernandez@academicos.udg.mx (M.R.H.-P.); alfonso.gomez@academicos.udg.mx (H.A.G.-R.); jhonathan.cbedoya@academicos.udg.mx (J.C.-B.); 5 Facultad de Medicina y Ciencias Biomédicas, Universidad Autónoma de Chihuahua, Circuito Universitario 31109, Campus II, Chihuahua ZC 31512, CH, Mexico; lsilvas@uach.mx; 6 Laboratorio Clínico, Hospital Ángeles Andares, Calle Universidad 200, Zapopan ZC 45116, JA, Mexico; michelleledesma@gmail.com; 7 División de Genética, Centro de Investigación Biomédica de Occidente, Delegación Jalisco, Instituto Mexicano del Seguro Social, Sierra Mojada 800, Colonia Independencia, Guadalajara ZC 44340, JA, Mexico; ingriddavalos@hotmail.com; 8 Departamento de Patología Clínica y Molecular, Instituto NIDIAC, Cobalto 2, Ciudad Industrial, Durango ZC 34208, DG, Mexico; nidiac@prodigy.net.mx

**Keywords:** pre-eclampsia, *AGT* gene, haplotypes, *AGT* polymorphisms, single nucleotide polymorphisms, SNPs, T174M, M235T

## Abstract

Pre-eclampsia is a Hypertensive Disorder of Pregnancy (HDP) characterized by hypertension and proteinuria, affecting 2–8% of pregnancies worldwide and constituting a major public health concern. Genes of the renin–angiotensin system have been investigated as potential causative factors, but inconclusive results have been obtained. The objective of this pilot study is to evaluate the possible contribution of alleles, genotypes or haplotypes of two single-nucleotide polymorphisms (SNPs) T174M (rs4762) and M235T (rs699) in *AGT* gene to pre-eclampsia in the Mexican population. We analyzed the association by performing PCR-RFLP with DNA extracted from whole blood samples of Mexican women with pre-eclampsia or normotensive pregnancy and the general population (GP). Our results showed a significant difference in the rate of heterozygosity for the T174M polymorphism between cases and controls. In addition, this polymorphism together with homozygosity for the M235T polymorphism may represent a possible genetic marker associated with pre-eclampsia. The T-C haplotype (174M–M235) was more common in patients with pre-eclampsia (non-significant difference *p* = 0.0503). The identification of genetic risk markers may support the early detection of pre-eclampsia and strengthen peripartum maternal health strategies within a global health framework aimed at reducing maternal mortality.

## 1. Introduction

Pre-eclampsia is classified as a hypertensive disorder of pregnancy (HDP) and is characteristically marked by the new onset of hypertension accompanied by proteinuria, most commonly arising after 20 weeks of gestation. According to the American College of Obstetricians and Gynecologists (ACOG), proteinuria is established by any of the following: (1) ≥300 mg of protein in a 24 h urine specimen; (2) a protein-to-creatinine ratio ≥ 0.3 mg/dL; or (3) a dipstick measurement of 2+ (when quantitative assessments are unavailable). Notably, pre-eclampsia may also be diagnosed in the absence of proteinuria when other clinical abnormalities are present, including: thrombocytopenia, defined as a platelet count < 100,000 × 10^9^/L; hepatic dysfunction, reflected by transaminase concentrations exceeding twice the upper limit of normal; severe right upper quadrant or epigastric pain not attributable to alternative conditions; renal insufficiency, indicated by serum creatinine > 1.1 mg/dL or a doubling of baseline creatinine without underlying renal pathology; pulmonary edema; or new-onset, treatment-resistant headache or visual disturbances unrelated to other diagnoses. Pre-eclampsia with severe features is identified when blood pressure reaches a systolic value ≥ 160 mm Hg and a diastolic value ≥ 110 mm Hg on two separate measurements at least four hours apart. The constellation of hemolysis, elevated liver enzymes, and thrombocytopenia—commonly referred to as HELLP syndrome—constitutes a severe variant of pre-eclampsia, typically presenting in the third trimester and associated with heightened maternal morbidity and mortality. Eclampsia is diagnosed when symptoms of hyperreflexia, seizures, or coma occur in a patient with a prior diagnosis of pre-eclampsia [1,2].

Pre-eclampsia represents a public health problem worldwide. The World Health Organization estimates that approximately 70,000 maternal deaths related to hypertensive complications of pregnancy occur annually, with a higher prevalence in low- and middle-income countries [3]. Despite advances in obstetric management and decades of research, the ability to predict these disorders in a timely manner remains limited.

The pathophysiology of pre-eclampsia includes defective trophoblastic invasion, endothelial dysfunction, and an exacerbated immune response [4,5]. These processes may be mediated by genetic factors, opening the possibility of identifying predictive markers, making clinical laboratory testing essential for the timely diagnosis of this condition. Some polymorphisms in the angiotensinogen gene and other genes of the renin-angiotensin system have been associated with high blood pressure and may influence the risk of pre-eclampsia [6,7,8,9].

The renin–angiotensin system (RAS) is a hormonal signaling mechanism that regulates blood pressure. The classical pathway begins with the release of renin (REN), an aspartyl protease that cleaves angiotensinogen (*AGT*) to produce angiotensin I (AngI), which is subsequently hydrolyzed by angiotensin-converting enzyme 1 (ACE) to produce angiotensin II (AngII). This octapeptide exerts its functions (primarily vasoconstriction) through specific receptors, the angiotensin II receptor type 1 and type 2 (*AGTR1* and *AGTR2*, respectively) [10].

Multiple previous studies have suggested that variants of the *AGT* gene are associated with increased plasma levels of *AGT*, and may contribute to the hereditary components of predisposition to high blood pressure [11,12,13,14,15]; however, several authors have reported that the association of variants in the *AGT* gene with hypertension has not been conclusive, due to the limited number of studies, as well as the heterogeneity and variability in the genetic and phenotypic makeup of the populations studied [6,7,16,17].

The *AGT* gene is located at 1q42-43, comprising five exons and four introns spanning 13 kb. Multiple polymorphisms have been described, but interest has focused on two of them, missense polymorphisms, located in the coding region: M235T (rs699) and T174M (rs4762), both located in exon 2 [18]. The objective of this study was to describe the distribution of alleles, genotypes, and haplotypes of the T174M and M235T polymorphisms of the *AGT* gene in patients with pre-eclampsia from Northwest Mexico.

## 2. Materials and Methods

In total, 177 genomic DNA (gDNA) samples were analyzed: 77 from Mexican mestizo individuals from the general population of Northwest Mexico (men and women) in whom Hardy–Weinberg equilibrium (HWE) of both polymorphisms was determined; 100 samples obtained over a two-year period from Mexican mestizo women who were at or after the 20th week of gestation, of which 49 were assigned to the case group and 51 controls over 20 years of age, including users of a tertiary-level public hospital, who participated in a previous project and gave their informed consent specifying that they accept that, once the original project was completed, their DNA sample would be kept anonymous and could be used for the analysis of other genetic markers. The project was approved by the Local Research Committee 1301 of the Mexican Social Security Institute with registration number R-2009-1305-3, dated 7 August 2009. Women with previous diagnosis of chronic hypertension, diabetes mellitus, gestational glucose intolerance, ischemic heart disease, dyslipidemia or nephropathy hypertension or proteinuria prior to week 20 of gestation were not included in none of the study groups, as well as those with multiple pregnancy. Pre-eclampsia was defined as the presence of hypertension and proteinuria in accordance with the criteria of the report of the National High Blood Pressure Education Program [19].

### 2.1. Genotyping of AGT Polymorphisms

gDNA was extracted from peripheral blood leukocytes by using standard protocols [20,21]. DNA concentration was determined by spectrophotometry using the NanoDrop system (ThermoFisher Scientific Inc. Waltham, MA, USA). Genotypes for both polymorphisms were identified using PCR-RFLPs protocols described previously [8]. Negative and positive controls were included in all PCR reactions.

### 2.2. T174M (rs4762)

In total, 100 ng of gDNA was amplified for 35 cycles in a reaction volume of 10 µL containing 0.8 pmol/µL of primers 5′-TGGCACCCTGGCCTCTCTCTATCT-3′ (forward) and 5′-CAGCCTGCATGAACCTGTCAATCT-3′ (reverse), 2 mM MgCl_2_, 0.25 mM of each dNTP and 0.75U Taq DNA polymerase. The 165-bp amplicon was exposed to the restriction enzyme *TthIII I* for 16 h at 65 °C and electrophoresed on a 8% polyacrilamide gel (29:1) with silver nitrate staining to identify the genotypes C/C, C/T and T/T.

### 2.3. M235T (rs699)

In total, 100 ng of gDNA was amplified for 35 cycles in a reaction volume of 10 µL containing 0.8 pmol/µL of primers 5′-CAGGGTGCTGTCCACACTGGACCCC-3′ (forward) 5′-CCGTTTGTGCAGGGCCTGGCTCTCT-3′ (reverse), 1.5 mM MgCl_2_ 0.25 mm of each dNTP and 0.75U Taq DNA polymerase. The 303-bp amplicon was exposed to the restriction enzyme *NcoI* for 16 h at 65 °C and electrophoresed on a 8% polyacrilamide gel (29:1) with silver nitrate staining to identify the genotypes C/C, C/T and T/T.

Haplotypes were designated in this study with the nucleotide present in each allele in the following order: T174M-M235T.

### 2.4. Statistical Analysis

Quantitative variables were expressed as means ± standard deviations (SDs), and qualitative variables were expressed as frequencies and percentages (%). Comparisons of proportions between groups were computed using the chi-square test and between means using the independent sample *t*-test. The Hardy–Weinberg equilibrium (HWE) in the control subjects was determined by comparing the observed and expected data using the chi-square test. A *p*-value was considered significant if *p* ≤ 0.05. Arlequin software version 2007 (University of Bern, Bern, Switzerland) was used to infer haplotypes and assess the Hardy–Weinberg equilibrium.

## 3. Results

### 3.1. Patients

This study included 49 patients with pre-eclampsia (cases) and 51 women with normotensive pregnancy (control group); recruited in a previous study. All patients were recruited from the pregnancy care unit of an obstetrics and gynecology hospital, where routine monthly prenatal check-ups are conducted. Pre-eclampsia was defined as the presence of hypertension and proteinuria. Clinical assessment included measurements of blood pressure, height, and body weight. Blood pressure was measured on two occasions, 6 h apart, using a mercury sphygmomanometer on the patient’s right arm; systolic and diastolic pressures were determined based on the first and fifth Korotkoff sounds, respectively. Body mass index (BMI) was calculated as weight in kilograms divided by height in meters squared (kg/m^2^) [22]. Their demographic, anthropometric, clinical, and biochemical characteristics, obtained from their medical histories, are described in Table 1. Differences were found in the number of cesarean sections, use of antihypertensive drugs, and systolic and diastolic blood pressure.

### 3.2. Alleles and Genotypes

In a total of 51 women with normotensive pregnancies, genotypic and allelic distributions of both polymorphisms were analyzed. For the M235T polymorphism, genotyping was successfully performed in 49 cases, whereas for the T174M polymorphism, 40 cases of pre-eclampsia were successfully genotyped (Table 2). No deviations from Hardy–Weinberg Equilibrium were detected in either of the two polymorphisms (*p* ≥ 0.05).

### 3.3. Combinations of Genotypes

Table 3 shows the genotype combinations in the study population. Two of the genotype combinations were not found in any of the subjects, and one combination was found in only one of them. Significant difference in the distribution of T174M C/T in combination with M235T T/T between the two groups was found.

### 3.4. Haplotypes

The haplotypes were inferred from the combinations of genotypes present. Five patients in the normotensive pregnancy group and five in the pre-eclampsia group were shown to be double heterozygotes; that is, they are heterozygous in both polymorphisms. They were thus eliminated from the haplotype analysis because it was not possible to define the phase. Table 4 shows only the haplotype data derived from genotypes that were identified in more than one individual across all groups, except for the double heterozygotes. The T-C haplotype showed a trend towards statistical significance, occurring more frequently in women with pre-eclampsia.

## 4. Discussion

Maternal care accounts for approximately 40% of medical and surgical interventions performed in Mexico. It is estimated that up to 80% of maternal deaths are preventable with risk-based prenatal care. Complications are difficult to predict, but it has been estimated that 46.4% of maternal deaths are related to professional negligence and 9.7% to hospital negligence; 15% of pregnancies may become complicated and require significant obstetric intervention. The link with other factors is noteworthy: diabetes (10.3%) and hypertension (18.4%) in women ≥ 20 years of age, and pregnancy in women < 20 and >35 years of age for the health of newborns [23].

Advances in omics sciences (genomics, transcriptomics, metabolomics, etc.) have enabled the identification of genetic variants and expression profiles that correlate with an increased risk of pre-eclampsia. Techniques such as ELISA and qRT-PCR, among others, allow for the quantification of biomarkers in biological fluids. Sequencing and genotyping are used to identify polymorphisms or alterations in gene expression in blood or placental tissue samples [24,25,26,27,28]. The identification of genetic risk markers represents a promising strategy for the early detection of pre-eclampsia. Clinical application still requires extensive validation, but its development could mark a turning point in reducing maternal mortality. Therefore, medical tools that allow for improved early diagnosis of pre-eclampsia before the third trimester of pregnancy will offer multiple benefits to the mother and her fetus. Blood pressure and proteinuria have proven to be less accurate than other biomarkers. The current recommendation is to combine biomarker measurements with all available information in a multi-marker modeling approach for the timely detection of adverse conditions in women with suspected disease [13].

In the Mexican population, polymorphisms in genes other than those of the renin–angiotensin system have been analyzed in women with pre-eclampsia. Machorro et al. analyzed the *PstI* and *MaeIII* variants of the insulin gene; the *NsiI* variant of the insulin receptor gene; and the Ala513Pro and Gly972Arg polymorphisms of the insulin receptor substrate 1 gene, without finding an association with this hypertensive disorder of pregnancy [22]. More than three decades ago, it was recognized that molecular variants of the *AGT* gene can constitute hereditary predispositions to hypertension in humans [29], and it has been confirmed that the M235T and T174M polymorphisms are independent predictors of early-onset pre-eclampsia in the Romanian population, for example [14]. The allelic and genotypic frequencies of both polymorphisms, described in women with normotensive pregnancies from other populations, are similar to those found in the Mexican population [8,12,18,30,31,32,33]; however, in women with pre-eclampsia, as in our study, there are significant differences, particularly in the heterozygous genotype of the T174M polymorphism, which confers a predisposition to pre-eclampsia [15]. The association of the alternative allele of this polymorphism (174M) with hypertension has already been demonstrated in the Mexican population [34], and, moreover, the risk of developing pre-eclampsia is increased in women whose genotypes contain alternative alleles with a possible pathological biological effect, as is the case with the genes for angiotensinogen, endothelial nitric oxide synthase, and guanine binding protein [35]. Although the functional mechanism by which this allele increases blood pressure in carriers is not well elucidated, the high frequency of the reference allele (T174) in all populations and the fact that Threonine at this position is highly conserved among hominids suggests a positive evolutionary selection of this polymorphism [29].

We found a statistically significant difference in the frequency of the C/T and T/T genotype combination between women with normotensive pregnancy and with pre-eclampsia (Table 3); however, as the small sample size limits our ability to interpret the results, future studies are needed to determine its prognostic value further.

The frequency of the T174M and M235T haplotypes of the *AGT* gene has been analyzed in women with pre-eclampsia from other populations [14,34] with no significant differences compared to our results (*p* > 0.05). The T-C haplotype (174M-M235) showed differences bordering on statistical significance between women with normotensive pregnancies and those with pre-eclampsia in the studied population (*p* = 0.0503). The sample size is a limitation of our study, and it is likely that studying a larger number of patients would allow us to define the biological significance of this haplotype in relation to the risk of pre-eclampsia, considering that it contains the alternative allele of the T174M polymorphism, which has been associated with high blood pressure in carriers.

The genetic constitution of the Mexican population is unique, due to its historically mixed and highly diverse origin, the result of complex demographic processes that occurred mainly from the period of conquest and colonization, with very deep pre-Hispanic antecedents and European and African contributions, which gave rise to a genetically complex, structured mixed population that is distinct from other Latin American or worldwide populations; therefore, our results are important as part of a personalized medicine approach. This study provides an approximation of the genetic makeup of the Mexican mestizo population in relation to polymorphisms in the *AGT* gene, and in this context, the need for further studies with larger sample sizes should be considered. Furthermore, women with a history of pre-eclampsia have an increased risk of cardiovascular disease and should receive risk reduction counseling [36], hence the need to identify predictive markers for this condition and for pre-eclampsia with sufficient prognostic value that can be translated into clinical practice [37]. The methodology for identifying the two most studied polymorphisms of the *AGT* gene is simple and relatively inexpensive, which highlights the need for clinical laboratories to implement this type of genomic testing to expand knowledge of the genetic makeup of populations and identify genetic risk markers, which represents a promising strategy for the early detection of pre-eclampsia and the reduction in maternal mortality from this cause.

## 5. Conclusions

Our results, while limited due to the sample size, show that the heterozygous genotype for the T174M polymorphism in combination with the alternative homozygous genotype for the M235T polymorphism could be a genetic marker associated with pre-eclampsia in Mexican patients. Although the differences did not reach statistical significance, the T-C haplotype (alternative allele for T174M polymorphism) is probably associated with pre-eclampsia in women from Northwest Mexico, which will need to be confirmed in a larger number of women.

## Figures and Tables

**Table 1:** Clinical characteristics of women with normotensive pregnancy or pre-eclampsia.

Characteristic	Normotensive Pregnancy (*n* = 51)	Pre-Eclampsia (*n* = 49)	*p*
Age, years	25.9 ± 5.9	26.04 ± 5.416	0.940
Parity, *n*	0.9 ± 1.9	0.67 ± 1.34	0.398
Miscarriages, *n*	0.2 ± 0.6	0.2 ± 0.7	0.898
Cesarean section, *n*	1.0 ± 0.9	0.5 ± 0.7	0.012
Antihypertensive drug, *n* ^a^	0	1.28 ± 0.9	<0.001
Weight, kg	61.8 ± 12.3	59.4 ± 8.1	0.298
Height, m	1.6 ± 0.1	1.5 ± 0.1	0.523
BMI	24.4 ± 4.5	23.7 ± 3.1	0.393
Systolic blood pressure, mmHg	114 ± 9	134 ± 14	<0.001
Diastolic blood pressure, mmHg	75 ± 7	85 ± 11	<0.001
Glucose, mg/dL	73.8 ± 14.3	74.5 ± 10.8	0.787
Insulin, µU/mL	8.0 ± 7.3	6.7 ± 6.8	0.399
HOMA-IR	1.6 ± 1.74	1.2 ± 1.2	0.071

Values are expressed as means ± SDs. ^a^ Treatment with alpha-methyldopa and/or hydralazine. HOMA-IR: Homeostatic Model Assessment for Insulin Resistance.

**Table 2:** Genotype and allele frequencies of T174M and M235T polymorphisms in *AGT*.

Polymorphism	Genotype or Allele	Normotensive Pregnancy	Pre-Eclampsia	*p*
		*n* = 51	*n* = 40	
T174M (rs4762)	C/C	44	26	0.3836
	C/T	6	14	0.0348
	T/T	1	0	-
	C	94	66	0.6137
	T	8	14	0.0802
		*n* = 51	*n* = 49	
M235T (rs699)	C/C	6	3	0.3674
	C/T	32	22	0.3265
	T/T	13	24	0.0984
	C	44	27	0.1111
	T	58	70	0.3148

**Table 3:** Genotype combinations of T174M and M235T polymorphisms in *AGT*.

Combination of Genotypes		Normotensive Pregnancy (*n* = 51)	Pre-Eclampsia (*n* = 40)
T174M	M235T		
C/C	C/C	6	2
C/C	C/T	26	12
C/C	T/T	11	11
C/T	C/C	0	1
C/T	C/T	5	5
C/T	T/T	2	9 *
T/T	C/C	0	0
T/T	C/T	1	0
T/T	T/T	0	0

* Significant difference (*p* = 0.02).

**Table 4:** Haplotype distribution in the study groups (T174M/M235T).

Haplotype (Amino Acids)	Haplotype (Nucleotides)	Normotensive Pregnancy	Pre-Eclampsia	*p*
TM	C-C	38	17	0.133
TT	C-T	50	42	0.6176
MT	T-C	3	8	0.0503
Total		91	67	

## Data Availability

The original contributions presented in this study are included in the article. Further inquiries can be directed to the corresponding author.
